# Reviewing the Traditional/Modern Uses, Phytochemistry, Essential Oils/Extracts and Pharmacology of *Embelia ribes* Burm.

**DOI:** 10.3390/antiox11071359

**Published:** 2022-07-13

**Authors:** Vineet Sharma, Dev Nath Singh Gautam, Andrei-Flavius Radu, Tapan Behl, Simona Gabriela Bungau, Cosmin Mihai Vesa

**Affiliations:** 1 Department of Rasa Shastra & Bhaishajya Kalpana, Faculty of Ayurveda, Institute of Medical Sciences, Banaras Hindu University, Varanasi 221005, India; vinitbhu93@gmail.com; 2 Doctoral School of Biomedical Sciences, Faculty of Medicine and Pharmacy, University of Oradea, 410073 Oradea, Romania; andreiflavius.radu@gmail.com; 3 Department of Preclinical Disciplines, Faculty of Medicine and Pharmacy, University of Oradea, 410073 Oradea, Romania; v_cosmin_15@yahoo.com; 4 Department of Pharmacology, Chitkara College of Pharmacy, Chitkara University, Punjab 140401, India; tapanbehl31@gmail.com; 5 Department of Pharmacy, Faculty of Medicine and Pharmacy, University of Oradea, 410028 Oradea, Romania

**Keywords:** *Embelia ribes* Burm., Vidanga, antioxidants, essential oils/extracts, embelin, vilangin, pharmacology, phytochemistry, plant-based compounds

## Abstract

Objectives: *Embelia ribes* Burm. (*E. ribes*, Myrsinaceae), also known as Vidanga in Ayurveda, has been shown to have significant therapeutic benefits on several disorders, and its main chemical bioactive constituent, embelin, has the therapeutic potential to be converted into innovative drugs, which is why it has recently received considerable interest. In the present work, we provide a higher level of comprehension, awareness, and extensive knowledge of the traditional uses, phytochemistry, and pharmacological characteristics of *E. ribes* throughout the last several decades (February 1965 to June 2021), emphasizing the importance of the study of essential oils extracted from *E. ribes*, which show a major potential for exerting antioxidant and anti-inflammatory activity. Materials and Methods: Google Scholar, MEDLINE, EMBASE, Scifinder, Scopus, and ScienceDirect were used to conduct a thorough literature search. Results: *E. ribes* is high in essential oils, alkaloids, flavonoids, steroids, and phenolics, all of which have medicinal benefits. The essential oils/extracts and isolated chemical constituents exhibited antioxidant activity, wound healing, antidiabetic, central nervous system (CNS)-related disease, antiviral, antiobesity, cardioprotective, antifungal, antibacterial, and antifertility activity, among other promising pharmacological effects. Conclusion: The translation between traditional applications and modern medicine may make *E. ribes* a promising target for the implementation of innovative medication. To investigate the efficacy and safety profile of *E. ribes*, further high-quality preclinical studies using advanced methodologies are required.

## 1. Introduction

*Embeliaribes* Burm. (*E. ribes*) is a woody shrub from the family Myrsinaceae, commonly known as Vidanga, and it has been used in traditional medicine due to several observed effects, including analgesic, anthelmintic, antioxidant, antibacterial, antidiabetic, anticancer, antihyperlipidemic, wound healing, and anti-spermatogenic activity, etc. [1,2]. It is a plant widely distributed in Cambodia, south China, India, Laos, Malaysia, Sri Lanka, Thailand, Vietnam, etc. In the mature fruits, the globular berries are dark red to almost black in color, with a tiny beak-like protrusion at the apex and five-lobed persisting calyxes. The epicarp′s thin epidermis emerged warty, and the middle wide mesocarp is composed of large tabular parenchymatous tissues, widely dispersed groups of stony cells and fibrovascular bundles. Simple, druse crystals of calcium oxalate and starch grains (elliptical-shaped) were also identified. Layers of brachy sclereids with a pyramidal shape make up the endocarp. Endocarp containing a single seed is surrounded by stony endocarp. The seeds′ bases are depressed inward incursions of the perisperm and ruminating endosperm, and they are mottled with yellowish brown dots [3].

Embelin (with IUPAC name 2,5 dihydroxy-3-undecyl-1,4 benzoquinone) is an important bioactive substance found in the *Embelia* genus. Hepatoprotective, anti-inflammatory, antioxidant, antimitotic, radioprotective, anticancer, contraceptive, anti-spermatogenic, anti-infective, antihyperlipidemic, antihyperglycemic, analgesic, antipyretic, and wound healing activity are included in embelin′s therapeutic profile [4].

Traditional Chinese medicine includes ethnic medicine as an important component. Due to their consistent therapeutic results and low clinical toxicity, ethnic medicine and its formulations have gained wide acceptance in Asian and Western countries in recent years. Therefore, considerable research is focused on the evaluation of ethnic remedies. *E. ribes* exhibits a variety of biological actions, among the most promising of which are its anti-tumor, antioxidation, and anti-inflammatory properties, according to pharmacological examinations [5].

The literature on this topic can be described as insufficiently developed, with few published papers focusing exhaustively and extensively on *E. ribes* (as it can be also seen from the number of the final references mentioned in the PRISMA diagram), which led to the idea of conducting this research. The main purpose of our work is to provide up-to-date and in-depth information on the traditional uses, phytochemistry, and pharmacology of *E. ribes*, documented over the years, up to the present. In addition, research into the therapeutic potential of essential oils/extracts and isolated compounds obtained from *E. ribes*, correlated with their antioxidant potential, has highlighted the scientific connotation of traditional uses and described the value and importance of considering both contemporary therapeutic known actions and traditional uses in folk medicine.

## 2. Methodology

**Figure 1:**
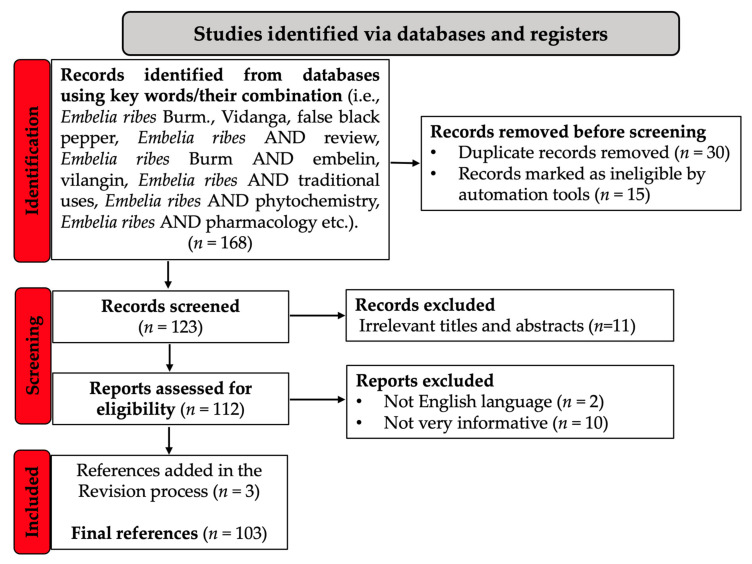
PRISMA flow chart describing the process of published data selection.

To perform a review of the pharmacological activity of *E. ribes*, emphasizing the antioxidant potential of the essential oils, its traditional usage, and phytochemistry, a thorough literature search was conducted. Between February 1965 to June 2021, we used scientific electronic databases, including SCiFinder, PubMed, MEDLINE, EMBASE, Scopus, ScienceDirect, and the Google Scholar library; medical subject heading (MeSH) terms were used for searching in PubMed. The keywords were searched alone and in conjunction, using the Boolean operator AND. The PRISMA flow chart describes in Figure 1 the methodology for the already published information selection, using the directions given by Page et al. [6,7].

## 3. Traditional Uses

**Figure 2:**
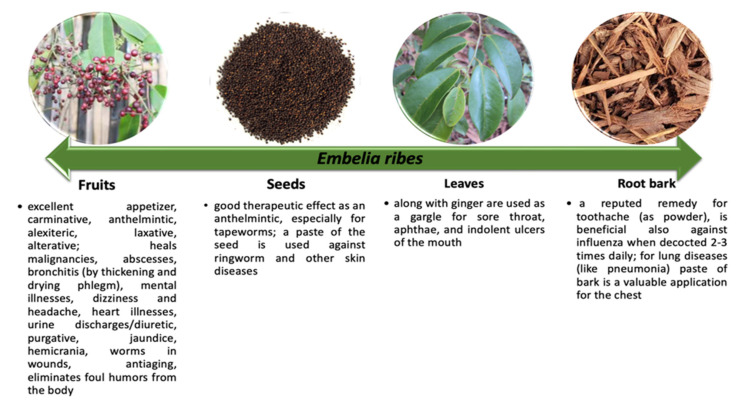
Summarizing the main effects of different parts of *E. ribes* plant.

**Figure 3:**
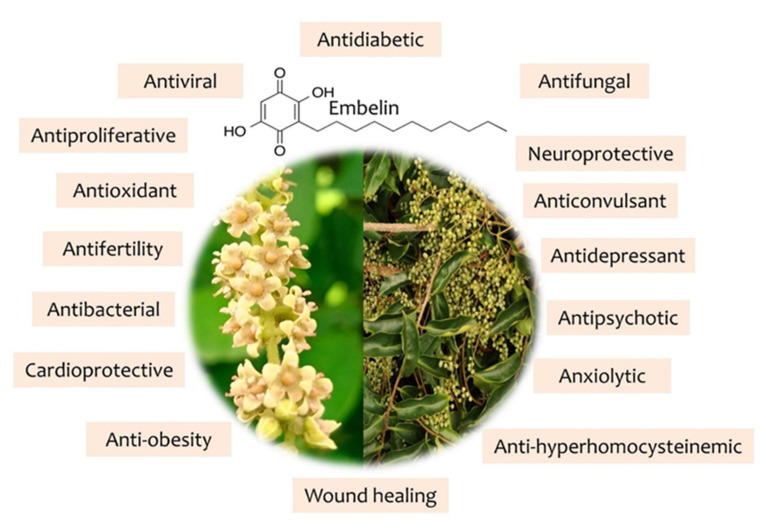
Pharmacological activities induced by embelin.

Traditional, ancient medicine was plant based, also using parts of animals and different substances from nature, being a mix between religion and science [8]. In this regard, different parts of *E. ribes* were used for their therapeutical actions [3,9,10] as is presented in Figure 2, and the pharmacological activity due to embelin is highlighted in Figure 3.

For more than 5000 years, *E. ribes* has been utilized traditionally for a variety of medical conditions. Traditional applications also varied according to the part of the plant that is used (e.g., fruits, seeds, root barks, leaves). Ancient uses suggested various forms of application (e.g., paste, powder, oil, and decoction). The paste was used for mouthwashes in preventing cavities, but also for skin-related disorders; the powder was used for various types of infections, indigestion, constipation, epilepsy, and as a blood purifier; the oil was used for dermatologic disorders and wound infections, and decoction of the roots was administered in cardiac diseases and insanity. Moreover, *E. ribes* has also been used for rejuvenation, bloating, vomiting, gastritis, and was frequently used in weight loss therapy and as a contraceptive. Among the most common effects obtained in traditional uses are carminative, anti-malignant, diuretic (e.g., fruit), anthelmintic (e.g., seeds), antibacterial, and pneumoprotective (e.g., leaves, root bark) [11,12].

Traditional uses have been translated to modern medicine and different pharmacological effects have been evaluated through experimental studies in order to elucidate mechanisms of action, dosages, precautions etc. Among the phytocompounds with the greatest potential are embelin, vilangin, embeliaflavosides and embelialkylresorcinols.

## 4. Essential Oils/Extracts and Nanoparticles of *E. ribes*

### 4.1. Aqueous Extract

Several experimental studies have been conducted in order to assess the correlation between the phytochemistry and pharmacology of *E. ribes*’ phytocompounds, the importance of essential oils progressively increased with the development of analytical methods.

In 2006, scientists, mostly from the Department of Pharmaceutical Botany at Hamdard University, New Delhi, India, botanically authenticated dried fruits of *E. ribes* (voucher specimen no. UB 2) purchased at a local market. The output of the aqueous extract of *E. ribes* was about 5.261% on average. Since previous research indicated the *E. ribes* extracts efficiency in doses of 100 and 200 mg/kg b.w., the resulted aqueous extract of *E. ribes* was diluted in Tween 80 and provided orally to adult male Wistar albino rats [13]. Standardization of *E. ribes* aqueous extract was performed by using standard chemical tests, used to conduct preliminary phytochemical screening of *E. ribes* fruits’ aqueous extract, for phytoconstituents detection. The extract contained essential oils, alkaloids, proteins, flavonoids, carbohydrates, phenolic components, and saponins, these compounds being considered responsible for its antioxidant action [13].

### 4.2. Embelin Isolation Method

The plant was procured from a local market located in Delhi, India. The berries of *E. ribes* were air dried, finely pulverized, and preserved in an airtight container. After the extraction with n-hexane using the Soxhlet equipment, the solvent was evaporated via distillation. The residue was further mixed with cold pet ether. Furthermore, the residue was then homogenized in a solution of methanol and dichloromethane (DCM), which was stored to crystallize. After crystallization, the particles were collected by filtration and washed firstly with n-hexane, then with DCM. The embelin was obtained in a yellowish colored crystalline structure described by morphology, coloration, and consistency using analytical techniques such as infrared (IR), high-performance thin-layer chromatography (HPTLC), and liquid chromatography–mass spectrometry (LC-MS) [14].

### 4.3. Ethanolic Extract

The leaf ethanol extract was used to isolate the phytocompound embelin. On a silica gel chromatographic column, the ethanolic extract was evaluated and eluted with methanol and chloroform. Thin layer chromatography (TLC) was utilized to analyze the eluted fractions. Furthermore, they were divided into five fractions. The higher-concentration determined in fraction two was recrystallized from chloroform to produce an orange red needle-like aromatic molecule. Quinones were qualitatively examined in the crystalline compound. IR and proton nuclear magnetic resonance (^1^HNMR) were used to confirm the structure. The ethanolic extracts and the phytocompound embelin were used to generate two different types of drug formulations (e.g., 0.2% gel for topical application and oral suspension of 30 mg/mL of crude ethanolic extracts) [15].

### 4.4. Gold and Silver Nanoparticles

Green synthesis of gold (GNPs) and silver (SNPs) nanoparticles has stimulated the interest of researchers in the subject of nanomedicine in recent years. The seed extract of *E. ribes* (SEEr) was used as a capping and reducing agent, which resulted in an eco-friendly, cost-effective, fast, and simple technique for the synthesis of GNPs and SNPs. Ultraviolet-to-visible (UV-Vis) spectroscopy, dynamic light scattering (DLS), high-resolution transmission electron microscopy (HR-TEM), Fourier transform infrared (FT-IR), and X-ray powder diffraction (XRD) were used to characterize the synthesized GNPs and SNPs. The α,α-diphenyl-β-picrylhydrazyl (DPPH) and phosphomolybdenum assays were used to determine the ability of GNPs and SNPs to scavenge free radicals. Furthermore, the antibacterial activity of GNPs and SNPs against two microorganisms was measured using the disc diffusion method, and the cytotoxicity of GNPs and SNPs against MCF-7 cell lines at different doses was determined using the 3-[4,5-dimethylthiazol-2-yl]-2,5 diphenyl tetrazolium bromide (MTT) assay. Both GNPs and SNPs developed from *E. ribes* produced promising results, demonstrating their clinical significance [16].

UV-Vis spectrophotometer examination revealed the synthesis of GNPs and SNPs from a solution of silver nitrate and chloroauric acid. Due to the reduction of metal ions to neutral, the gold solution has changed from light yellow to wine red, with a specific peak in the region between 500 and 550 nm, whilst the silver solution has changed from colorless to yellowish-brown, with a peak between 400 and 440 nm.

Alkaloids, quinones, proteins, reducing sugars, and saponins, among other elements of *E. ribes* seed extract, may be responsible for metal ion reduction. SEEr-GNPs and -SNPs were 10–30 nm in size and 5–35 nm in size, respectively. GNPs and SNPs developed from SEEr are spherical and polydisperse in nature. SEEr-GNPs had a hydrodynamic diameter of 6 to 68 nm, while SEEr-SNPs had a diameter of 5 to 122 nm [16].

### 4.5. Vilangin

Even though certain well-known bioactive principles such as embelin have been comprehensively examined, the presence of other low polar and volatile components, as well as their bioactivities, has yet to be investigated. Furthermore, *E. ribes* fruits also contain an alkaloid (i.e., christembine) and a volatile oil in addition to embelin (i.e., vilangin) [17].

The antioxidant and anticancer properties of vilangin, a dimeric embelin derivative synthesized from embelin and isolated from *E. ribes* berries, were recently discovered in experimental studies [18].

**Figure 4:**
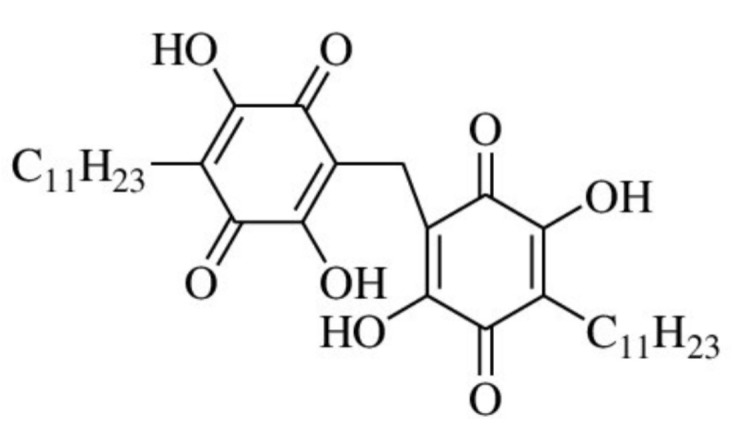
Structure of vilangin.

Vilangin is synthesized by mixing embelin and formaldehyde in an acetic acid solution. By gradually warming, embelin was dissolved in glacial acetic acid. Moreover, formaldehyde was added, and the solution was heated on a water bath, then followed the cooling process to room temperature. The intense orange precipitate was filtered and crystallized from dioxane. The structure of vilangin is depicted in Figure 4. The IUPAC name is 2-[(2,5-dihydroxy-3,6-dioxo-4-undecylcyclohexa-1,4-dien-1-yl)methyl]-3,6-dihydroxy-5-undecylcyclohexa-2,5-diene-1,4-dione [17,18].

Several experimental studies were conducted in order to test the antioxidant potential (i.e., DPPH assay, cupric ion reducing antioxidant capacity assay, ferric reducing antioxidant power assay, and total antioxidant capacity) and anticancer activity (i.e., cytotoxicity assessment) of vilangin, which has been less studied [17,19].

Vilangin′s antioxidant activity was evaluated and compared to a standard (butylated hydroxytoluene). At 1000 µg/mL, the radical scavenging activity was at its highest (72.35). Cupric ion decreasing antioxidant capacity of vilangin was based on concentration. At a concentration of 1 mg/mL, the reference had a higher cupric ion decreasing antioxidant ability than vilangin. In the presence of antioxidants, the ferric reducing antioxidant capacity assay measures the reduction of ferric iron Fe(3+) to ferrous iron Fe(2+). Moreover, the assay is extensively used to determine single antioxidant activity and total antioxidant activity in plant extracts [18].

At 40 µL, the activity was comparable to that of conventional butylated hydroxytoluene. When compared to butylated hydroxytoluene (2.206 ± 0.11 mM Fe(II)/g), vilangin (1.084 ± 0.02 mM Fe(II)/g) had an almost twofold superior ferric reducing capacity [17].

The phosphomolybdenum approach was used to assess vilangin′s total antioxidant activity. This approach relies on the antioxidant substances reducing molybdenum Mo (VI)–Mo (V) and forming a green Mo (V)-antioxidant complex with a maximum absorption at 695 nm. Vilangin (0.842 ± 0.01 mg gallic acid equivalent/g) and butylated hydroxytoluene 1.137 ± 0.06 mg gallic acid equivalent/g) exhibited high absorbance values, indicating that the compounds showed an important antioxidant activity [18].

It has been reported that vilangin possessed cytotoxic effect against the A549 lung adenocarcinoma cancer cell line in vitro. It showed 61.95% activity at a dosage of 500 µg/mL, with an IC50 of 400 µg/mL (53.66%). In a concentration-dependent way, all of the concentrations employed in the experiment reduced cell viability significantly (*p* < 0.05).

Further studies are needed to establish the efficacy and safety profile of vilangin, an antioxidant phytocompound less investigated than embelin [17,18].

## 5. Phytochemistry of Bioactive Compounds

According to the scientific literature, the following substances were isolated from *E. ribes*:

- Three chemical compounds, identified as embelin (1), embeliaribyl ester (2), embeliol (3), and embelinol (4) were isolated from the seeds [20];
- 3-alkyl-1,4-benzoquinone (5), N-(3-carboxylpropyl)-5-amino-2-hydroxy-3-tridecyl-1,4-benzoquinone (6), o-methyl rapanone (7) and rapanone (8) were isolated from the plant [21];
- Embelialkyl resorcinols A-I (9–17), virenol A (18), pentaketide (19), 1-(3,5-dihydroxyphenyl)heptan-1′-one (20), 1-(3,5-dihydroxyphenyl) nonan-1′-one (21), and 1-(3,5-dihydroxyphenyl)undecan-1′-one (22–26) were isolated from the ethanolic fruits extract [22];
- N-(3-carboxylpropyl)-5-amino-2-hydroxy-3-tridecyl-1,4-benzoquinone (27), 5,6-dihydroxy-7-tridecyl-3-[4-tridecyl-3-hydroxy-5-oxo-2(5H)-furylidene]-2-oxo-3(2H)-benzofuran (28), 2,5-dihydroxy-3-tridecyl-1,4-benzoquinone (29), 9 2,5-dihydroxyl-3-undecyl-1,4-benzoquinone (30), 10 2,5-dihydroxyl-3-pentadecyl-1,4-benzoquinone (31), 9 5-(8Z-pentadecenyl)-1,3-benzenediol (32), 11 5-(8Z-heptadecenyl)-1,3-benzenediol (33), 12 5-(8Z,11Z-heptadecadienyl)-1,3-benzenediol (34), 13 5-pentadecyl-1,3-benzenediol (35), 12 3-methoxy-5-pentylphenol,14 3,5-dimethoxy-4-hydroxy phenyl-1-O-β-D-glucopyranoside (36), 15 2,6-dimethoxy-4-hydroxyphenyl-1-O-β-D-glucopyranoside,16 (37) (+)-catechin (38), 17 and (+)-lyoniresinol-3α-O-β-glucoside (39), sitosterol (40) and daucosterol (41) were isolated from the ethanolic extract of the roots [23,24];
- Five phenolic derivatives with various aliphatic chains and two phenolic glucosides, identified as 5-(8-pentadecenyl)-1, 3-benzenediol (42), 5-(8, 11-heptadecadienyl)-1, 3-benzenediol (43), 5-pentadecyl-1, 3-benzenediol (44), 5-(8-heptadecenyl)-1, 3-benzenediol (45), 3-methoxy-5-pentane-1-phenol (46), 3, 5-dimethoxy-4-hydroxyphenyl-1-O-β-D-glucopyranoside (47), and 2, 6-dimethoxy-4-hydroxyphenyl-1-O-β-D-glucopyranoside (48) respectively were isolated from the plant [24];
- Three flavonoid glycosides, embelia flavosides A-C (49–51) were isolated from the fruits [25];
- A new alkenyl resorcinol compound (namely embeliphenol A) was isolated from ethyl acetate stems extract [26].

**Figure 5:**
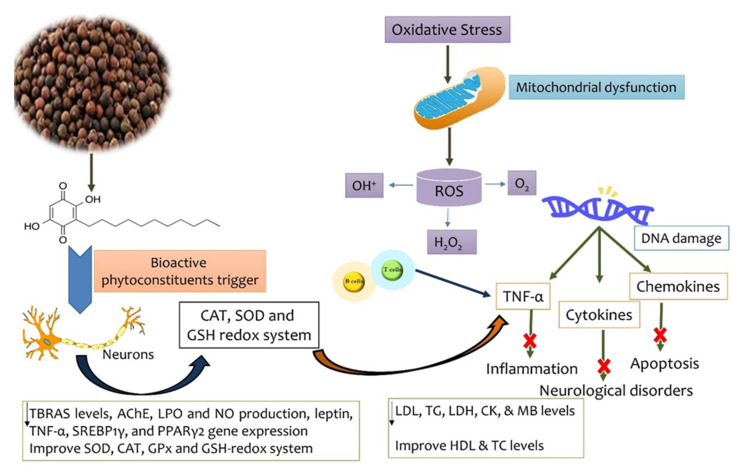
Mechanism of action of *E. ribes* and its bioactive compounds. ROS, reactive oxygen species; H_2_O_2_, hydrogen peroxide; DNA, deoxyribonucleic acid; TNF-α, tumor necrosis factor-alpha; CAT, catalase; SOD, superoxide dismutase; GSH, glutathione; LDL, low density lipoprotein; TG, triglyceride; LDH, lactate dehydrogenase; CK&MB, creatin kinase iso-enzyme; HDL, high density lipoprotein; TC, total cholesterol; TBRAS, thiobarbituric acid reactive substance; AchE, acetylcholine esterase; LPO, lactoperoxydase; NO, nitric oxide; SREBP1γ, sterol regulatory element-binding proteins 1 gamma; PPARγ2 peroxisome proliferator-activated receptor gamma 2; GPx, glutathione peroxidase.

The activity of the plant-based compounds identified above is shown in Figure 5, given that their chemical composition and structure imprint their interactions, as well as their multiple chemical, biochemical, physical, and pharmacological properties [27].

## 6. Pharmacological Properties

### 6.1. Wound Healing Activity

Ethanol leaf extract (30 mg/mL) and embelin (4 mg/mL) isolated from *E. ribes* were evaluated for wound healing activity in albino rats. The wound healing activity of ethanol extract and embelin was found significant as compared with framycetin (standard skin ointment). The tested drug showed faster epithelialization of wounds with a high rate of wound contraction, higher tensile strength, and collagenation. The histopathological study exhibited improved cross-linking of collagen fibers and the absence of monocytes [15].

### 6.2. Antidiabetic Activity

Diabetes mellitus (DM) has been treated orally with natural remedies, based on folk medication, since ancient times. The Indian classical text of Ayurveda describes *E. ribes* as pungent, a therapeutic tool that produces an improvement in digestive fire, healing flatulence, and colic. Bhandari et al. presented the first pilot study concerning biochemical confirmation of the potential of *E. ribes* in diabetic dyslipidemia. The lipid-lowering and antioxidant activity of ethanol extract (200 mg/kg, p.o., for 20 days) of *E. ribes* were studied in streptozotocin (STZ)-induced (40 mg/kg) diabetes in rats. The ethanol extract treated diabetic rats and exhibited a significant (*p* < 0.01) reduction in blood glucose, serum total cholesterol, and triglycerides, with elevated HDL cholesterol levels as compared with STZ-induced diabetic rats. The ethanol extract reduced the thiobarbituric acid-reactive substances (TBARS) values of the liver and pancreas as compared to TBARS values of the liver and pancreas of STZ-induced diabetic rats. The results of the test drug were found to be similar to gliclazide (25 mg/kg, p.o.), a standard antihyperglycemic drug [28].

In another study, embelin (25 and 50 mg/kg, p.o., for 21 days) was administered to alloxan-induced diabetic rats, which showed a decrease in fasting serum blood glucose levels and an increase in the rat′s body weight. The histological examination of the liver, kidney, and pancreas showed that normal architecture confirmed that embelin recovered the biological function of the liver, kidney, and pancreas [29]. In addition, embeliphenol A showed α-glucosidase inhibitory potency in a concentration-dependent manner [26].

Nephropathy linked with type 2 diabetes is the most prevalent cause of the end-stage renal disorder. The protective effect of ethanolic fruit extract of *E. ribes* (100 and 200 mg/kg, p.o., for 21 days) was evaluated against a high-fat diet and low dose STZ (35 mg/kg, i.p.)-induced diabetic nephrotoxicity in rats. Ethanolic fruit extracts of *E. ribes* displayed protective effects by a significant decrease (*p* < 0.01) of body weight, fasting blood glucose level, blood pressure (BP), alkaline phosphatase, serum lactate dehydrogenase, total cholesterol, creatinine, and triglycerides, whereas a rise in serum albumin and level of total protein was also detected. Furthermore, ethanolic fruit extracts of *E. ribes* treatment significantly (*p* < 0.01) reduced the kidney TBARS levels and elevated catalase (CAT), glutathione (GSH), and superoxide dismutase (SOD) levels in diabetic rats [30].

Aqueous extracts of *E. ribes* (100 and 200 mg/kg, p.o. for 40 days) alleviated renal damage in STZ-induced diabetic rats, by improvement in blood glucose, lipid metabolism, BP-lowering, inhibition of pancreatic lipid peroxidation process, and increased the levels of pancreatic SOD, CAT, and GSH [31]. Oral administration of ethanolic extracts of *E. ribes* (100 and 200 mg/kg, for 6 weeks) showed a significant reduction in the blood sugar levels, glycated hemoglobin, heart rate, and systolic BP in rats as compared to diseased rats [32].

The antidiabetic activity of ethanolic extract of *E. ribes* (100 and 200 mg kg/day, for 21 days) was performed in a high-fat diet and low dose STZ-induced diabetic rats. Antihyperglycemic potency of ethanolic extract of *E. ribes* was exhibited by significant reduction (*p* < 0.01) in blood sugar levels, hepatic glucose-6-phosphatase effect, and an elevated content of glycogen. Moreover, the ethanolic extract significantly (*p* < 0.01) reinstated the increased BP, and slowed down the degeneration of liver fat and oxidative alterations in diabetic rats [33]. A 50% ethanolic extract of berries of *E. ribes* reduced blood glucose levels in alloxan-induced diabetic rats [34].

The efficacy and safety profile of *E. ribes* and its active biomarker, embelin, in the treatment of DM were assessed in a systematic review and meta-analysis. The inverse-variance model was used to conduct a meta-analysis of *E. ribes*/embelin/derivatives of embelin versus diabetic control. The systematic review and meta-analysis comprised a total of 13 research studies, all of which were conducted on rat models. Blood glucose and glycosylated hemoglobin were considerably reduced by *E. ribes* extracts and embelin. Moreover, the results of the meta-analysis also revealed significant improvements in insulin and lipid profile, hemodynamic parameters, and oxidative stress markers. Two embelin derivatives that also relieved diabetes symptoms were 6-bromoembelin and vilangin. The therapeutic potential of *E. ribes* helped to reduce the effects of diabetes-related weight gain. The medical data supports *E. ribes*/embelin/embelin derivatives′ anti-diabetic effectiveness. However, more clinical trial research is needed to confirm the current findings [35].

### 6.3. Anti-Obesity Activity

The anti-obesity potential of standardized ethanol extract of *E. ribes* (100 mg/kg, p.o., for 21 days) was evaluated in murine model of high-fat diet (HFD)-induced obesity. Standardized ethanolic extract displayed a preventive activity on the gain of body weight, accumulation of visceral fat, and higher BP. Moreover, standardized ethanolic extract of *E. ribes* treatment reduced the myocardial lipid peroxidation (LPO) and improved antioxidant levels in obese rats [36].

The preventive activity of embelin (50 mg/kg, p.o., for 21 days) was evaluated against hyperlipidemia and oxidative stress in HFD-induced obesity in male Wistar rats. To induce obesity, animals were fed with an HFD for a period of 28 days. Administration of embelin reduced body weight, BP, visceral fat pad weight, and lipid levels. In addition, embelin significantly (*p* < 0.01) reduced the level of hepatic TBARS, but elevated the level of CAT, SOD, and GSH levels in obese animals [37]. Aqueous extracts of *E. ribes* improved insulin resistance in a rat model with HFD-induced obesity, the possible mechanism of action being the downregulation of leptin, tumor necrosis factor-alpha (TNF-α), sterol regulatory element-binding proteins 1 gamma (SREBP1γ), and peroxisome proliferator-activated receptor gamma 2 (PPARγ2) gene expression. The leptin may contribute to hepatic steatosis by promoting insulin resistance and by altering insulin signaling in hepatocytes, which consequently promote increased intracellular fatty acid [38].

Embelin reduced adiposity in C57BL/6 mice by reducing the oxidative stress and inflammation caused by the HFD. Oral treatment of embelin resulted in a significant decrease in levels of nuclear factor erythroid 2-related factor and nuclear factor kappa-B (NF-κB) protein expression in liver tissue, as well as an improvement in obesity biomarkers. Thiobarbituric acid reactive substance (TBRAS), GSH, SOD, and CAT levels in liver tissue are also improved in embelin-treated HFD-fed mice along necrotic and inflammatory alterations that were significantly reduced in the liver tissue [14].

### 6.4. Cardioprotective Activity

The cardioprotective potential of *E. ribes* water fruits has been assessed in a rat model of acute myocardial infarction produced by isoproterenol (ISO) (5.25 and 8.5 mg/kg, s.c., for 2 days) and ISO-induced cardiomyopathy in STZ (40 mg/kg, i.v.)-induced diabetic rats. Water *E. ribes* extract (100 mg/kg, p.o., for 40 days) and alcoholic extract (200 mg/kg, p.o., for 40 days) lowered heart rate, systolic BP, elevated serum lactate dehydrogenase (LDH), serum creatine kinase (CK), and myocardial LPO, and markedly improved myocardial endogenous antioxidants (GSH, SOD, and CAT) levels [39,40,41].

### 6.5. Antioxidant Activity

A hydroxyl group is considered beneficial when examining radical scavenging properties since it releases a H atom, rendering the scavenged radical less reactive. It can also generate a quinone structure if an ortho or para-H-atom is available for abstraction, as in the case of the 3,4-dihydroxyphenyl moiety of quercetin. Embelin, on the other hand, is unable to react with superoxide in the classical manner, as recently demonstrated by structural and computational investigations [42].

Embelin scavenges the superoxide radical by extracting its electron and releasing molecular oxygen in a distinctive manner. The fact that both embelin hydroxyl groups form strong intramolecular H-bonds with the carbonyl groups in the surroundings, makes them less available for scavenging. Furthermore, embelin′s ability to protect against paraquat-induced lung damage was investigated in correlation to its antioxidant and anti-inflammatory properties. Malondialdehyde (MDA), SOD, CAT, glutathione peroxidase (GPx), inflammatory cytokines (i.e., interleukin-1β, TNF-α, interleukin-6) and NF-κB/mitogen-activated protein kinase (NF-κB/MAPK) were evaluated in experimental assays on lung tissue. Embelin therapy reduced MDA while increasing SOD, CAT, and GPx levels. In paraquat-administered and paraquat-intoxicated rats, embelin significantly lowered inflammatory cytokine levels. The results showed the impact of embelin′s activity on the oxidative effects of paraquat [43,44].

Embeliaflavosides A–C (flavonoid glycosides) showed significant 2,2′-azino-bis(3-ethylbenzothiazoline)-6-sulphonic acid (ABTS) radical scavenging potency (IC_50_ = 2.52–9.78 µM), and DPPH scavenging activity (IC_50_ = 7.56–26.47 µM) [25]. The antioxidant activity of ethanolic and aqueous extracts of *E. ribes* flower was investigated in vitro by determining nitric oxide (NO) scavenging activity, ferric thiocyanate, and total reduction efficiency, as well as by DPPH assays. Ethanolic and aqueous extracts of *E. ribes* flower showed a concentration-dependent escalation in NO, DPPH free radical, ferric thiocyanate inhibition/scavenging action, and equivalent overall reduction capacity. Compared to aqueous extracts, ethanolic extracts displayed greater scavenging action [45].

The scavenging capability of embelin has been demonstrated by utilizing hydrodynamic voltammetry, which produces the superoxide radical in situ. Embelin as a scavenger of superoxide outperforms the conventional food additive antioxidant 2,6-bis(1,1-dimethylethyl)-4-20methylphenol (butylated hydroxytoluene). Moreover, in the voltaic cell, embelin can even completely eliminate the superoxide radical. According to computational results, two types of embelin scavenging actions may be implicated, the first by π–π interaction mechanisms and the second through proton capture in the cell [2,46].

The biological activity of THP-1 human leukemic monocytes and BV-2 mouse microglia was examined in a study to assess its antioxidant properties. The antioxidant effects examined in MTT assay, proliferation curves, and antioxidant activity using a fluorescent probe showed promising results after 24 h. The long alkyl C10 tail of embelin may be essential for cell membrane insertion, which promotes the antioxidant defense mechanism and cytoprotection in microglia. Consequently, embelin may be a promising pharmaceutical tool for reducing the damage caused by metabolic and neurodegenerative illnesses [46].

*E. ribes* is a traditional Chinese herbal medicine that is used to treat a variety of ailments. However, there is no solid information on its chemical composition. The components of *E. ribes* were examined using ultra-high-performance liquid chromatography quadrupole time-of-flight tandem mass spectrometry (UHPLC-Q-TOF mass spectrometry). Numerous compounds were detected, including 16 phenolics, 16 flavonoids, 5 fatty acids, and 4 coumarins [2].

The total phenolic and total flavonoid content of the acetic ether extract of *E. ribes* was also analyzed. The results showed a major potential of *E. ribes* to be an important source of phenolics (308.16 mg gallic acid equivalents/g of extract) and flavonoids (62.00 mg rutin equivalents/g of extract). Furthermore, acetic ether extract had a significant antioxidant effect (ferric reducing activity power: 0.15 mg/mL; DPPH: 0.18 mg/mL; ABTS: 0.06 mg/mL). Acetic ether extracts also inhibited NO production in lipopolysaccharide (LPS)-stimulated macrophage RAW 264.7 cells and the release of pro-inflammatory cytokines. These results support the hypothesis that *E. ribes* can be an effective antioxidant and anti-inflammatory agent [5].

#### Pulse Radiolytic Assessment

The dynamics and molecular processes of embelin′s reactivity with hydroxyl (•OH), one-electron oxidizing, peroxyl, and thiyl radicals were examined using nanosecond electron pulse radiolysis to better understand the mechanism for its antioxidant function. Because embelin is poorly soluble in neutral water solutions due to intramolecular hydrogen bonding, investigations in alkaline conditions in aqueous solutions have been conducted [47,48].

At pH 10, embelin interacts with •OH to produce a broad transitory absorption spectrum in the wavelength range of 380–480 nm that lasts up to 100 µs. The reactivity of embelin with one-electron oxidants, such as azidyl (N_3_•) and trichloromethyl peroxyl radicals (•CCl_3_O_2_), has also been investigated in order to separate phenoxyl type radical absorption bands from OH-adduct and transitory due to H-abstraction. Adducts and phenoxyl type radicals are expected to be produced when embelin (O_2_E(OH)_2_) interacts with •OH and oxidants, respectively. The large absorption band in the 380–480 nm area can be attributed to transient conditions produced by OH-addition and/or H-abstraction, according to a comparison of transitory absorption spectra seen in its interaction with •OH to those recorded with one-electron oxidants [47].

Due to significant intramolecular hydrogen bond, radiolysis of embelin in non-polar solvents such as CH_2_Cl_2_ and 1-chlorobutane did not result in an oxidation reaction. A comparison of transient absorption data at 410 nm also reveals that only 35% of the •OH undergoes one-electron oxidation, with the rest forming •OH adducts on the ring or H-abstraction on the alkyl side chain. Furthermore, as related to the N_3_• radical reaction, 85% of the •CCl3O2 radical induces one-electron oxidation [49].

The medical importance of embelin requires research into its interactions with other free radicals of biological significance, as well as the regeneration of embelin free radicals using a regularly used antioxidant, ascorbic acid. Thiyl radicals (RS•) are formed during cellular redox processes and as a result of GSH′s antioxidant effect. These are reactive oxidants that target lipids, causing them to oxidize or isomerize. As a result, thiyl radical repair is required for the storage of thiols (RSH) for antioxidant action and to protect lipids [50,51].

The absorption spectrum of embelin with glutathiyl radical (GS•) at pH 3.9 is comparable to that of phenoxyl radical, with peaks at 430 and 320 nm. With GS•, however, there is no time resolution in the absorption spectrum. This indicates that GS• only produces its phenoxyl type radical when it combines with embelin [52].

Embelin has been discovered to scavenge biologically relevant oxidizing radicals significantly. Embelin scavenged •OH and GS• to form embelin phenoxyl radical (λ_max_ 410 nm), which is then scavenged by ascorbate anion. In vitro research shows that it can inhibit lipid peroxidation, reduce Fe(3+), and restore Mn-SOD, indicating that it could be an effective antioxidant in the biological system. Its free radical scavenging activity has also been reported to be superior to that of α-tocopherol. It can be assumed that, in addition to its metabolic functions, embelin′s antioxidant activity is due to its therapeutic properties [47].

### 6.6. Neuroprotective Effect

Elevated plus maze (EPM) and Morris water maze model (MWM) were used to test the anti-Alzheimer’s activity of embelin versus diazepam (1 mg/kg, b.w., i.p.)-induced amnesia. In the EPM model, embelin reduced the transfer latency time in a dose-dependent way, while in the MWM model, it reduced the escape latency time. The diazepam-treated group exhibited substantial increases in escape and transfer delay, indicating impairment in learning and memory. On the other hand, embelin significantly reversed the diazepam-induced amnesia and enhanced learning and memory in mice models in a dosage and time-dependent way [53].

Clinically recognized targets of Alzheimer′s disease (AD) include beta-secretase (BACE-1) and cholinesterase, both of which have benefited greatly from natural products [54]. Considering AD is a significant public health concern, it needs the use of multi-targeted medications to treat it [55,56]. The enzymes namely acetylcholinesterase (AChE), butyrylcholinesterase (BChE), and β-site of amyloid precursor protein cleaving enzyme (BACE-1) were inhibited by embelin, with IC50 values of 2.5, 5.4, and 2.1 μM, respectively. Embelin also increased the activity of P-gp, an efflux pump implicated in the clearance of amyloid-beta from the AD brain, in LS-180 cells. In addition, a study of cell viability in a neural cell line revealed that embelin had a wide therapeutic profile. These results suggested that embelin is a multi-targeted drug that reduces amyloid oligomer formation through BACE-1 inhibition [57], enhances cholinergic transmission through AChE/BChE inhibition, and promotes amyloid clearance (through P-gp induction) [58].

Antioxidants have been studied in the addition to developing neuroprotective medicines that could be used in stroke treatment. Stroke is an acute and progressive neurological condition that is the second largest cause of death. Ethanolic and aqueous fruits extracts of *E. ribes* (100 and 200 mg/kg, p.o., for 30 days) increased antioxidant activity and demonstrated neuroprotective potential against middle cerebral artery occlusion (MCAO)-induced localized cerebral ischemia in male Wistar rats. When compared to MCAO+ vehicle group rats, ethanolic and aqueous fruits extract of *E. ribes* gradually improved grip strength behavior, GSH, GPx, glutathione reductase (GR), and glutathione-S-transferase (GST) levels in the brain, as well as a significant decline in LDH levels in serum and TBARS levels in the brain [13,59]. As compared to ischemia control, pretreatment with embelin (25 and 50 mg/kg, p.o.) enhanced locomotor activity, holding latency time, and falling beam walking latency. Embelin decreased LPO and enhanced total thiol content and GST activity [60].

### 6.7. Anxiolytic Activity

The anxiolytic effect of embelin was evaluated in EPM and open field test (OFT) models. In the EPM model, embelin significantly improved the percentage of time spent and the number of entries. Embelin increased the time spent, crossing number, and reduced the time of immobility in the lightbox. Embelin showed significant growth in the rearing number, assisted rearing, and squares crossed in OFT [61].

The anxiolytic effect of the methanolic extract of *E. ribes* (100 and 300 mg/kg p.o.) was examined using the hole board test, EPM, mirrored chamber equipment, and gamma-aminobutyric acid (GABA) estimation. The number of entries and time spent in the open arm of EPM was increased by methanolic extract of *E. ribes*. In the hole board test, methanolic extract of *E. ribes* showed a substantial increase in the number of head dips. Furthermore, methanolic extract of *E. ribes* exhibited an increase in the number of entries and time spent in the mirrored chamber, as well as a reduction in the time it took to enter the mirrored room. GABA concentration in the brain was significantly increased by methanolic extract of *E. ribes* at the oral dose level of 300 mg/kg [62].

According to Afzal et al., embelin possesses strong anxiolytic action that is dose dependent. It has been hypothesized that the observed activity was due to an antagonistic effect on the GABA receptor complex, since most anxiolytic and antidepressant drugs selectively bind to the GABA receptor′s high-affinity benzodiazepine binding region. A small dark safe compartment and a large lighted preference chamber compose the test apparatus. In the EPM device, embelin at a dose of 5 mg/kg and embedelin (2.5 mg and 5 mg/kg) significantly enhanced the percentage of time spent and the number of entries in the open arm. In the EPM-test on mice, the results showed that embelin had dose-dependent anxiolytic action. In an open field experimental test, embelin showed a considerable rise in the number of crossing, rearing, and assisted rearing [61].

### 6.8. Antidepressant Activity

Under the tail suspension test (TST) and forced swimming test (FST) experimental models, embelin (2.5 and 5 mg/kg, i.p.) showed therapeutic potential for controlling depression by reducing immobility in a dose-dependent manner. In both animal models, the efficacy of embelin (5 mg/kg) was found equivalent to the standard drug (i.e., imipramine, 15 mg/kg) [63].

The anti-depressant effect of embelin has been demonstrated in animal model experiments using two widely known assessment methods: TST and FST. In academic research, the TST is used to detect stress in rodents as an experimental approach. In mouse models, the FST is used to assess the anti-depressant efficacy of novel chemicals, anti-depressant agents, and experimental development targeted at translating or preventing depressive-like conditions. It is founded on the observation that an animal will become immobile if it is subjected to short-term continuous stress. It has been described as generating a circumstance in which the animal experiences “behavioral despair”, whereby the animal loses hope of surviving the stressful environment [52].

### 6.9. Antipsychotic Activity

Antipsychotic action of embelin (5 and 10 mg/kg, p.o., for 15 days) was investigated against stereotyped activity and apomorphine-induced climbing activity in rats and mice, respectively. Embelin inhibited apomorphine-induced climbing and stereotyped activity in rodents. In the brain of experimental animals, embelin also reversed increased levels of dopamine, noradrenaline, and serotonin neurotransmitters. In both animal models, embelin exhibited more substantial results at a dosage of 10 mg/kg [64].

### 6.10. Anticonvulsant Activity

Embelin (2.5, 5, and 10 mg/kg, i.p.) showed anticonvulsant activity by seizures inhibition, decrease in locomotion induced by electroshock, and pentylenetetrazole (PTZ) in a dose-dependent manner [65].

### 6.11. Antifertility Activity

Embelin, isolated from berries of *E. ribes*, altered the testicular histology and glycogen, gametogenic counts, and accessory sex gland fructose at the dose levels 0.3, 0.4 and 0.5 mg/kg body weight administered subcutaneously for 35 days [66].

### 6.12. Antibacterial Activity

Both Gram-positive and Gram-negative bacteria were used to investigate the antibacterial efficacy of embelin, an isolated component from *E. ribes* berries. The microdilution method and the agar plate method were used to investigate the minimal inhibitory and bactericidal concentrations of embelin. Embelin was shown to be bactericidal against Gram-positive bacteria and bacteriostatic against Gram-negative bacteria [67]. The antimicrobial activity of SEEr was evaluated against *Escherichia coli*, *Pseudomonas aeruginosa*, *Staphylococcus aureus*, and *Bacilus subtilis*. *Escherichia coli* exhibited the highest inhibition zone followed by *P. aeruginosa*, whereas *S. aureus* and *B. subtilis* both showed lower inhibitory zones [68].

### 6.13. Antifungal Activity

The SEEr showed inhibitory effects of the fungal growth, with the highest activity seen at 2.0 mg seed extract concentration. *Colletotrichum crassipes* displayed maximum inhibition zones, while *Cladosporium*, *Armillaria mellea*, *Colletotricum capsici*, *Aspergillus niger*, and *Rhizopus oryzae* were the next species tested for inhibition of the fungal growth. In comparison to other species, *Aspergillus terreus* and *Candida albicans* displayed smaller inhibitory zones after interaction with SEEr [69]. Furthermore, the standard in vitro antifungal susceptibility test techniques such as the national committee for clinical laboratory standard M27-A2 protocol (NCCLS) was used to assess the antifungal activity of various *E. ribes* extracts and its bioactive component, embelin. In 96 well plates, four different types of extracts (i.e., petroleum ether, solvent ether extract, methanol extract, and water extract) were tested, and detection was performed with a colorimetric plate reader at 530 nm. The NCCLS approach demonstrated that methanol extract and embelin showed the minimum inhibitory concentration required to inhibit the growth of 50% of organisms (MIC50) range of 120 mg/L against *Candida albicans* (MTCCno.183), and embelin had reported MIC50 values < 700 mg/L among the four *Candida* species studied. Except for the water extract, the percentage growth increased when the concentration of the plant extracts dropped [70].

### 6.14. Antiproliferative Activity

Embelin exhibited chemo-preventive activity against phenobarbital-induced hepatocarcinogenesis in Wistar rats. The crude hexane fruits extract of *E. ribes* demonstrated cytotoxicity toward human leukemic cells (K562) and Dalton′s lymphoma ascites (DLA) cells [71]. Embelialkylresorcinols C, E, F, and H showed moderate cytotoxicity (half maximal inhibitory concentration, IC50 = 23.06 to 41.49 μM) against three human cancer cell lines (i.e., Hep3B, A549, and HCC1806) [22]. The anticancer efficacy of embelin was determined using MCF-7 breast cancer cells in a cytotoxicity and apoptosis study. The IC50 value of embelin was found to be 80μg/mL in MCF-7 breast cancer cells. It was also shown that embelin was able to alter cell viability and trigger apoptosis in MCF-7 breast cancer cells in a dose-dependent way [72].

Rai et al. analyzed the survival time of cancer patients in the presence of various causes of mortality. The data of surveillance, epidemiology, and end results (SEER) were used to perform the study. According to the SEER program of the US National Cancer Institute (2000–2014), a total of 2875 people were diagnosed with breast cancer, with 577 of them dying as a result of the breast cancer disease [73]. In mice xenograft breast cancer models of MDA-MB-231 cells, embelin (10 mg/kg b.w., i.p. twice weekly for 28 days) reduced tumor volume and caspase-3 activation. Embelin triggers apoptosis via interacting with a variety of signaling pathways, which vary depending on the cancer′s origin. Embelin exhibited a variety of biological traits that are important to human cancer chemoprevention, and growing data suggests that embelin may regulate many tumor cell characteristics. NF-κB, tumor suppressor gene (p53), phosphoinositide 3-kinase/protein kinase B (PI3K/Akt), and signal transducer and activator of transcription 3 (STAT3) signaling pathways are all modulated by embelin, which causes cell apoptosis that can be intrinsic or extrinsic. Embelin stimulates autophagy in cancer cells, although these autophagic cell-death processes have received less attention than apoptotic cell-death processes [74].

The embelin effect on the development of human prostate cancer cells was studied. In comparison with the case of breast cancer (MCF-7, MDA-MB-231, and T47D), hepatoma (Hep3B, HepG2, and HuH-7), and choriocarcinoma (JEG-3), embelin significantly suppressed cell growth in human prostate cancer cell lines, including PC3, LNCaP-LN3, DU145 and normal prostate epithelial cell, RWPE-1. It has been discovered that embelin caused apoptosis in PC3 cells in a time-dependent approach, which was associated with decreased expression of B-cell lymphoma 2 (Bcl-2), B-cell lymphoma-extra-large (Bcl-xL), and myeloid cell leukemia 1 (Mcl-1), enhanced Bcl-2 associated x, apoptosis regulator (Bax) translocation into mitochondria, and a decrease in mitochondrial membrane potential [2].

Embelin increased the expression of the voltage-dependent anion channel (VDAC) 1 and oligomerization, which may stimulate cytochrome c and apoptosis-inducing factor (AIF) release. The effects on wingless-type (Wnt)/β-catenin signaling were investigated since embelin was able to decrease Akt activation and cyclooxygenase-2 (COX-2) production. Embelin inhibited β-catenin expression while activating glycogen synthase kinase (GSK)-3. In embelin-treated cells, there was a decrease in β-catenin-mediated T-cell factor (TCF) transcriptional activity and gene transcription, including cyclin D1, c-myelocytomatosis (c-myc), and matrix metalloproteinase (MMP)-7. Lithium chloride, a GSK-3 inhibitor, inhibited changes in β-catenin levels in response to embelin, suggesting that embelin may reduce β-catenin expression through GSK-3 activation. Moreover, when PC3 cells were exposed to embelin, cell migration and invasion were significantly reduced. In conclusion, these results imply that the pro-apoptotic impact of embelin in prostate cancer cells is partially mediated by suppression of Akt signaling and activation of GSK-3 [75].

The potential of embelin to provide a therapeutic effect on glioma was examined. Human glioma cells were discovered to be inhibited by embelin, although normal immortalized human astrocytes were not. In addition, embelin promoted apoptosis in human glioma cells by suppressing NF-κB, a critical transcription factor linked to a variety of human disorders, including cancer, that regulates a number of genes involved in tumor progression, including cell proliferation and survival. Even though embelin was found as an X-linked inhibitor of apoptosis protein (XIAP) inhibitor, it had no effect on XIAP in glioma cells, but instead reduced NF-κB activity by hindering the nuclear translocation of p65, as a result of decreasing phosphorylation and proteasomal degradation of nuclear factor of kappa light polypeptide gene enhancer in B-cells inhibitor, and alpha (IκBα) in glioma cells. Additionally, p65 overexpression in glioma cells reduced embelin-induced apoptosis. These results showed that embelin could be a promising new treatment for glioma by reducing NF-κB activity and thereby limiting cancer cell proliferation and inducing apoptosis [76].

Investigations seeking to define embelin′s precise molecular target resulted in the discovery of embelin as an inhibitor of the XIAP′s baculovirus inhibitory repeat domain (BIR3). In addition, embelin has been shown to inhibit 5-lipoxigenase (5-LOX) and microsomal prostaglandin E2 synthase-1 (mPGES-1), as well as plasminogen activator inhibitor-1 (PAI-1) and P300/CBP associated factor (PCAF). Furthermore, embelin has been observed to interfere with mitochondrial oxidative phosphorylation through both redox and non-redox processes [77].

Even though embelin has been shown to possess a wide range of therapeutic effects, the mechanisms by which it exerts anticancer benefits are as yet unclear. The critical role of oxidative stress-induced MAPK signaling as a primary mechanism for its anticancer effects has been discovered by tracking the molecular alterations associated with early apoptotic phase. Embelin treatment of A549 lung cancer cells resulted in an increase in phospho-p38 and phospho-c-jun n-terminal kinase (JNK) levels as early as 4 h. The activation of caspase-3 by embelin was blocked by pretreatment of cells with particular inhibitors of p38 (PD169316) and JNK (SP600125). The observed changes in phosphorylation levels of p38, JNK, and ERK 1/2 in the presence or absence of specific MAP kinase inhibitors are mainly attributable to embelin and not due to cross talk between MAP kinases, according to experimental studies. Pretreatment of cells with 5,10,15,20-tetrakis(N-methyl-4′-pyridyl) porphyrinato iron III (FeTMPyP) reduced embelin-induced changes in MAPK phosphorylation and apoptosis, indicating that reactive oxygen species (ROS) play a key role. The observations are not related to embelin′s inhibitory action on XIAP, as second mitochondria-derived activator of caspases N7 peptide (SMAC-N7-Ant peptide), a selective inhibitor of XIAP′s BIR3 domain, did not mimic embelin-induced apoptotic effects. The results of this study showed the significance of p38 and JNK pathways in embelin-induced apoptosis and provided scientific information for improving the therapeutic profile of embelin [78].

### 6.15. Antiviral Activity

Ethyl acetate fruits extract of *E. ribes* and the isolated compound embelin showed maximum antiviral potency with virus-inhibiting activity IC50 of 0.2 µg/mL, selectivity index (SI) = 32 and IC50 of 0.3 µM and SI = 10. Furthermore, embelin was tested against influenza viruses (i.e., influenza A and B viruses). The results of the experimental study showed that H5N2 virus was the most susceptible to embelin (SI = 31), whereas H3N2 virus had the greatest resistance (SI = 5) [79].

The antiviral activity of embelin (5, 10, and 20 mg/kg/day, i.p., for 4 days) was evaluated in LPS-induced acute respiratory syndrome in murine models. Pretreatment with embelin showed its potential as a therapeutic drug for acute respiratory distress syndrome by decreasing inflammation of the lung, mononucleated cellular infiltration, nitrate/nitrite, total protein, albumin concentrations, TNF-α in the bronchoalveolar lavage fluid, and myeloperoxidase activity in lung homogenate. Embelin protected the partial pressure of oxygen (pO_2_) down-regulation and the partial pressure of carbon dioxide (pCO_2_) augmentation [80].

In the human population, the herpes simplex virus-1 (HSV-1) causes a wide spectrum of infections, from minor to life-threatening. HSV-1 infections have effective therapies, although they are generally limited to HSV-1 latency and the development of resistance to current treatments. An experimental study investigated the impact of embelin on HSV-1 in cultivated Vero cells in terms of antioxidant and antiviral effects. A large amount of ROS (i.e., hydrogen peroxide, H_2_O_2_) was produced, indicating oxidative stress processes. Antiviral assays, antioxidant assays, penetration, and post penetration assays, confocal microscopy, quantitative polymerase chain reaction (qPCR), and time course attachment were performed after Vero cells were infected with a recombinant strain of HSV-1. The results of the conducted study showed that embelin is noncytotoxic at doses ranging from 20 to 70 µM. HSV-1 virions were treated with embelin, which inhibited the adhesion and penetration of HSV-1 virions to host cells, resulting in a 98.7–100% inhibition and affecting the early stages of HSV-1 infection in Vero cells. The production of H_2_O_2_ was substantially reduced when virions were treated with embelin concentrations ranging from 35 to 60 µM. Furthermore, embelin lowers oxidative damage induced by HSV-1 infection and is an efficient antiviral for reducing HSV-1 infection in Vero cells in culture. More research is needed to conclude if embelin may be used as a therapeutic agent [81].

### 6.16. Anti-Hyper Homocysteinemic Activity

The anti-hyper homocysteinemic and lipid-lowering potential of ethanol fruits extract (100 and 200 mg/kg, p.o., for 1 month) of *E. ribes* was studied in methionine (1 g/kg, p.o., for 1 month)-induced hyper homocysteinemia in male albino rats. Ethanol fruits extract significantly decreased levels of homocysteine, LDH, total cholesterol, triglycerides, LDL levels in serum, and LPO levels in homogenates of the heart, with an increase in serum HDL and myocardial GSH levels. All the findings of ethanol extract were compared with the standard drug (folic acid (100 mg/kg, p.o.)) [82]. Another study showed that aqueous fruits extract of *E. ribes* (100 and 200 mg/kg, p.o., for 1 month) significantly (*p* < 0.01) reduced the homocysteine levels, LDH, total cholesterol, triglycerides, LDL-C, and VLDL-C and elevated the HDL-C levels in serum. Furthermore, a significant (*p* < 0.01) reduction in LPO levels with improvement in GSH level was also found in hyper homocysteinemic rats [83].

### 6.17. Protection against Liver Injury

Embelin’s protective actions against acute liver injury have also been explored in the literature. Adult mice were administered a single injection of thioacetamide (TAA) (300 g/g body weight) to generate an animal model of acute liver damage. Embelin was given as a 50 g/g body weight intragastric gavage starting two days before TAA injection and continued throughout the research. The log-rank test was used to examine the mice′s survival using the Kaplan–Meier method. The acute liver injury procedure was repeated, and the mice were evaluated at the appropriate times. Necrosis/inflammation and liver regeneration were examined using hematoxylin and eosin staining and picrosirius red staining, respectively. The activity of serum alanine aminotransferase/alkaline phosphatase was used to measure liver function. Immunohistochemistry was used to assess the amounts of cleaved caspase-3 and F4/80 expression in the liver. GraphPad Software was used to conduct the statistical analysis to validate the experiment. The mice′s survival and liver function were significantly better in the group given embelin before TAA exposure than in the TAA exposure-only group. TAA-induced hepatic necrosis/apoptosis was diminished significantly by embelin. In the embelin-treated recovery group, massive inflammatory cell infiltration, which is associated with hepatic fibrogenesis, occurred earlier than in the spontaneous recovery group. Furthermore, embelin therapy boosted macrophage activity quickly and efficiently. Consequently, embelin may effectively prevent acute liver injury. Its therapeutic potential requires further investigation [84].

### 6.18. Anti-Aging

Embelin, a benzoquinone derivative with anticancer, anti-inflammatory, and antioxidative properties, has been investigated for potential anti-aging properties. However, little research has been conducted on the use of cosmetic raw components for embelin. Using a H_2_O_2_-induced cellular aging model of human dermal fibroblasts (HDFs), the antioxidant and anti-senescence properties of embelin were investigated. The water-soluble tetrazolium salt (WST-1) assay was used to determine cell viability. By using quantitative reverse transcription polymerase chain reaction (qRT-PCR), the gene expression model in HDFs by embelin has been quantified.

Changes in intracellular ROS concentration were assessed using dichlorofluorescein diacetate (DCF-DA). The senescence-associated β-galactosidase (SA-β-galactosidase) protocol, a method of staining β-galactosidase, was used to determine cell senescence. Cell mobility was measured using a wound healing technique. The WST-1 experiment revealed that embelin restored cell viability in a dose-dependent way after H_2_O_2_ reduced cell viability.

Furthermore, embelin elevated the expression of SOD1, GPx1, and CAT genes, implying that embelin-induced antioxidant potential may be strengthened by overexpression of intracellular antioxidant-related genes. The SA-β-galactosidase assay was used to examine whether embelin decreases cell senescence in an H_2_O_2_-induced senescence model of HDFs. Embelin reduced SA-β-galactosidase activity in H_2_O_2_-treated HDFs in a dose-dependent way. Moreover, embelin reduced the expression of the p21 and MMP1 genes in H_2_O_2_-treated HDFs in a dose-dependent approach. In H_2_O_2_-treated HDFs, however, embelin elevated COL1A1 genes in a dose-dependent manner. The study showed that embelin could be used as an anti-aging cosmetic component with anti-senescence and antioxidant characteristics [85].

### 6.19. Toxicological Profile

Experimental studies have demonstrated that not all herbal products are safe for direct human administration, especially in children and pregnant women, despite the fact that medicinal plants have less adverse effects than synthetic drugs. According to the results of the toxicity tests, administering rats and mice oral doses of embelin ranging from 10 mg to 3 g/kg was safe [86].

The spleen, kidney, and liver masses were unaltered by embelin administering in female cyclic rats at a dose of 120 mg/kg body weight, while the initial mass of the adrenals significantly increased. By administering embelin for 6 weeks, certain pathophysiological alterations can be observed. Whereas the spleen′s histological properties remained relatively constant, the adrenals exhibited enlargement. It has been documented that chicken model organisms given an oral dose of 1.25 g/kg of *E. ribes* experienced ganglion cell degeneration in their retinal cells. However, retinotoxicity and visual impairment have not been reported at a dose of 0.25 g/kg [2].

Two groups of pregnant rats were administered 2.5 times and 5 times the recommended dose of an Ayurvedic contraceptive known as pippalyadi vati, which contained equal portions of powdered seeds or fruit berries of *E. ribes*, fruit of *Piper longum*, and borax powder. The pregnant rats gave birth to shorter and lower birth weighted infants. Moreover, soft tissue abnormalities and intestinal herniation into the umbilical cord have been reported in the developing fetuses of pippalyadi-treated mothers. Similar herniation was not observed in the control groups [87].

The use of *E. ribes* should be monitored, especially when administered in higher doses for children and pregnant women, as experimental tests using these herbs in animal models have demonstrated potential toxicological effects.

## 7. Experimental and Clinical Studies

Experimental studies describing the effects of *E. ribes* Burm. on different types of cancer are summarized in Table 1.

Hypothyroidism is a hypometabolic clinical state resulting from inadequate production of thyroid hormones. Vidanga Vati was given thrice a day after meals with lukewarm water for the duration of 8 weeks. *E. ribes* showed statistically significant results on a 21.93% reduction in the level of thyroid-stimulating hormone-sensitive (s-TSH) and on almost all the signs and symptoms of hypothyroidism [102].

In children afflicted with ascarids, clinical trials using alcoholic and aqueous extracts of *E. ribes* fruits were conducted. Alcoholic extract was shown to be successful in 80% of instances, while aqueous extract was shown to be successful in 55% of cases, resulting in ova-free stools. No adverse effects were identified neither during nor after treatment. When the fruits of *E. ribes* were given to worm-infested individuals at dosages of 200 mg/kg, they had positive outcomes. In cases of infections with tapeworm, giardia, and nana, there was a significant improvement. It was enough to take a single dose of up to 8 g. Within 6 to 24 h after taking the medicine, the worms were ejected. The medicine was well tolerated and was considered to have a good safety profile [103].

## 8. Future Perspectives and Conclusions

As of now, the available medical literature indicates that after providing all the previous data, it can be concluded that *E. ribes* has been used to treat digestive, carminative, laxative, anti-helminthic, and other disorders as a traditional folk medicine in several Asiatic countries and cultures (India, Sri Lanka, Malaya, Singapore, and China). This review highlights the traditional usage, bioactive constituents, and pharmacological characteristics of *E. ribes* in order to explain the scientific connotation and boost the application of the medicinal value of *E. ribes*. Chemical components such as essential oils, alkaloids, phenols, and flavonoids abound in the plant, resulting as significant components with antioxidant, antidiabetic, anticancer, and other relevant therapeutical properties [2].

Embelin and vilangin are two natural compounds with major antioxidant potential, established by experimental studies, while further studies are needed to detail the molecular mechanisms and efficacy and safety profiles [1,18]. Consequently, *E. ribes* may become a useful medical tool in the future therapeutic management of numerous pathologies. Furthermore, the importance of chemical ingredients, current pharmacological effects, and classic usage in folk remedies are all detailed in the present paper. In-depth research on bioactive constituents, mechanisms, toxicity, pharmacokinetics, and clinical trials will be conducted in the future to give a more scientific understanding of *E. ribes*. These review′s findings will aid in the judicious use of *E. ribes* medication and its future development.

## Figures and Tables

**Table 1:** In vivo studies depicting the main pharmacological proprieties of *E. ribes*, administered as aqueous/ethanolic extract mainly from berries or other parts of the plant and as pure form.

Animal/Dose	Observation/Outcomes	Ref.
Anticancer		
AOM/DSS-induced colon cancer in C57BL/6 male mice/50 mg/d/kg b.w. mixed in diet for 10 days before the CAC challenge, then for 19 or 85 days	↓ Tumor incidence and volume, ↓ IL-6; ↓ STAT3	[88]
DENA/PB-induced liver carcinogenesis in Wistar male rats/50 mg/kg b.w. (p.o.), 14 weeks	↓ Neoplastic nodules	[89]
DMH models in C57 mice, both sexes/100 mg/d/kg b.w. mixed in diet for 30 weeks	↓ Tumor incidence and multiplicity, ↓ PCNA; ↓ Cox-2; ↓ c-Myc; ↓ Survivin	[90]
Female C57BL/6 with H7 or Panc 02 cells - ectopic mouse model/50 mg/kg b.w. i.p. daily, for 2 weeks - orthotopic mouse model/50 mg/kg b.w. i.p. every other day for 1 week	↓ Tumor volume and metastasis	[91]
Male Swiss albino mice solid tumor model with EAC cells/Photodynamic therapy with embelin, 12.5 mg/kg b.w. i.p.	↓ Tumor incidence and volume, ↓ myeloperoxidase, ↓ β-d-glucuronidase and Bcl-2; ↑ Rhodanese and Bax	[92]
**In disorders of the CNS**		
Swiss albino rats and mice/2.5, 5, and 10 mg/kg	Anticonvulsant: ↓ in the duration of HLTE in MES (2.5 and 5 mg/kg, i.p.) Electroshock,100% protection against mortality ↑ Clonic + tonic onsets at all doses	[65]
Swiss albino mice/2.5 and 5 mg/kg	Antidepressant-like effect in TST ↓ Immobility in the FST Exhibited significant activity in mice TST + FST experimental models	[63]
Male Wistar rats/50, 75, 100 mg/kg	Focal cerebral ischemia brain: ↓ infarction and edema (100 mg/kg) Decreased MDA level (75 and 100 mg/kg) ↑ SOD and CAT (100 mg/kg)	[93]
Swiss albino mice/2.5 and 5 mg/kg	Anxiolytic: ↑ Time spent and number of entries in open arm (elevated plus maze) ↓ Duration of immobility in light box (light/dark model) ↑ Rearing assisted rearing and number of squares crossed (open field test) Embelin: anxiolytic effect in dose-dependent manner	[61]
Male Swiss albino mice/10 and 20 mg/kg	Sickness: Embelin prevented anorexia, anhedonia, Ameliorated brain oxidative stress markers Protective effect in LPS-induced sickness behavior	[94]
Adult Wistar rats/10 and 20 mg/kg/day	Huntington′s disease: Loss of b.w. ↓ Oxidative stress ↓ 69–76% brain lesion Protect the neurons from 3-NP toxicity	[95]
Female C57BL/6 mice/25 and 50 mg/kg	Multiple sclerosis (autoimmune encephalomyelitis, CNS inflammation): ↓ Human CD14+ monocyte-derived dendritic cell differentiation ↓ Duction in the EAE clinical score. ↓ Inflammatory Th1 and Th17 cells in EAE.	[96]
Female Sprague–Dawley rats, male C57BL/6 mice/200 nM	Traumatic brain injury: Inhibition of NF-κB expression of XIAP increases in PFT-treated animals. p53 and NF-κB dependent mechanisms delayed neurodegeneration	[97]
Wistar rats’ pups/20 mg/kg	HI-induced neurological injury: Confirm sex differences in behavioral and anatomical outcome XIAP protect the female brain from the early HI injury	[98]
C57BL/6 male, GI female, and Ovx female mice/20 mg/kg	Cerebral ischemia: Inhibitor of XIAP exacerbated stroke-induced injury in females, no effect in males	[99]
Human glioma cell lines T98G, U87MG, and H4. IM-PHFA/0–50 μM	Apoptosis in human glioma cells via NF-κB inhibition: Embelin suppressed proliferation of human glioma cells Apoptosis in human glioma cells by inhibiting NF-κB. ↓ NF-κB activity by reducing nuclear translocation of p65	[76]
Human brain glioma U87 cells/0, 50, and 100 μg/mL	Apoptosis in human glioma cells via the mitochondrial pathway: Time- + dose-dependent apoptosis of brain glioma cells Arrest the cell cycle in the G0/G1 phase Changes in brain glioma cell mitochondrial membrane potential Shifting of Bax and Bcl-2 to cause apoptosis	[100]
Male Wistar rats/25 and 50 mg/kg	Global ischemia/reperfusion-induced brain injury: ↑ Locomotor activity and hanging latency time ↓ Beam walking latency ↓ Lipid peroxidation ↑ Total thiol content and glutathione-S-transferase neuroprotective agent and useful in the treatment of stroke	[60]
**Antidiabetics**		
Albino rats of either sex/100 and 200 mg/kg Wistar rats of either sex/200 mg/kg	Hemodynamic measurement (heart rate, systolic BP), blood glucose, HbA1c, blood GSH, serum marker enzymes (LDH and CK), oxidative stress markers in pancreatic tissue (SOD, CAT, GSH, and LPO), histopathology of pancreatic tissue	[31,41]
Wistar rats of either sex/200 mg/kg	Blood glucose, serum lipid profile (TC, TG and HDL), LPO and protein contents in liver and pancreas	[28]
Wistar rats of either sex/100 mg/kg	HbA1c, blood glucose and GSH, serum marker enzymes (CK, LDH), oxidative stress markers in pancreatic tissue (CAT, SOD, GSH, TBARS), histopathology exam of pancreatic tissue	[101]
Wistar albino rats of either sex/100 mg/kg	Hemodynamic measurement (systolic BP, heart rate), HbA1c, blood glucose	[32]
Male Wistar rats/100 mg/kg	Liver weight, b.w., fasting blood glucose, OGTT, hemodynamic measurement (systolic/diastolic BP, heart rate,); serum adiponectin, insulin, leptin, lipase levels; HOMA-IR values; hepatic glucose-6-phosphatase activity/glycogen content; serum lipid profile (AI, CRI, HDL, LDL, TC, TG, VLDL); oxidative stress markers in liver tissue (CAT, GSH, SOD, TBARS), histopathology exam of liver	[33]

3-NP, 3-nitropropionic acid; AI, atherogenic index; AOM, azoxymethane; ApoB, apolipoprotein B; Bax, Bcl-2 associated x, apoptosis regulator; Bcl-2, B-cell lymphoma 2; BMI, body mass index; BP, blood pressure; b.w., body weight; C57B6, C57 black 6; CAC, colitis-associated cancer; CAT, catalase; CK, creatine kinase; c-Myc, c-myelocytomatosis; CNS, central, nervous system; COX-2, cyclooxygenase-2; CRI, coronary risk index; DENA, Diethyl nitrosamine; DMH, 1,2-dimethylhydrazine; DSS, dextran sodium sulfate; EAC, Ehrlich’s ascites carcinoma; EAE, experimental autoimmune encephalomyelitis; FST, forced Swimming Test; GPx, glutathione peroxidase; GSH, glutathione; HbA1c, glycosylated hemoglobin; HDL, high-density lipoprotein; HFD, high-fat diet; H7, human embryonic stem cell (hESC) line 7; HI, hypoxia-ischemia; HLTE, hind limb tonic extension; HOMA-IR, homeostasis model assessment of insulin resistance; HPLC, high-performance liquid chromatography; IM-PHFA, immortalized primary human fetal astrocytes; IL-6, interleukin-6; i.p., intra peritoneal; ISO, isoproterenol; LDH, lactate dehydrogenase; LDL, low-density lipoprotein; LPO, lipid peroxidation; LPS, lipopolysaccharide; MDA, malondialdehyde; MES, maximal electroshock-induced seizure; NF-κB, nuclear factor kappa-light-chain-enhancer of activated B cells; NMR, nuclear magnetic resonance; NPD, normal pellet diet; OGTT, oral glucose tolerance test; PANC02, pancreatic adenocarcinoma epithelial cell line; p-Akt, phosphorylated protein kinase B; PB, phenobarbital; PCNA, proliferating cell nuclear antigen; PFT, pifithrin-alpha; p.o., per os; SOD, superoxide dismutase; STAT3, signal transducer and activator of transcription 3; STZ, streptozotocin; TBARS, thiobarbituric acid-reactive substances; TC, total cholesterol; TG, total triglyceride; TLC, thin layer chromatography; TNF-α, tumor necrosis factor-alpha; TST, tail suspension test; UV, ultraviolet; VLDL, very low-density lipoprotein; XIAP, X-linked inhibitor of apoptosis; ↓, decreasing/reducing; ↑, increasing.
